# Loss of Skeletal Mineralization by the Simultaneous Ablation of PHOSPHO1 and Alkaline Phosphatase Function: A Unified Model of the Mechanisms of Initiation of Skeletal Calcification

**DOI:** 10.1002/jbmr.195

**Published:** 2010-08-03

**Authors:** Manisha C Yadav, Ana Maria Sper Simão, Sonoko Narisawa, Carmen Huesa, Marc D McKee, Colin Farquharson, José Luis Millán

**Affiliations:** 1 Sanford Children's Health Research Center, Sanford-Burnham Medical Research Institute La Jolla, CA, USA; 2 Bone Biology Group, The Roslin Institute and Royal (Dick) School of Veterinary Studies, University of Edinburgh Edinburgh, UK; 3 Faculty of Dentistry, McGill University Montreal, Quebec, Canada

**Keywords:** OSTEOMALACIA, OSTEOIDOSIS, SCOLIOSIS, CALCIFICATION, BIOMINERALIZATION, HYPOPHOSPHATASIA, *AKP2*, TNAP

## Abstract

Endochondral ossification is a carefully orchestrated process mediated by promoters and inhibitors of mineralization. Phosphatases are implicated, but their identities and functions remain unclear. Alkaline phosphatase (TNAP) plays a crucial role promoting mineralization of the extracellular matrix by restricting the concentration of the calcification inhibitor inorganic pyrophosphate (PP_*i*_). Mutations in the *TNAP* gene cause hypophosphatasia, a heritable form of rickets and osteomalacia. Here we show that PHOSPHO1, a phosphatase with specificity for phosphoethanolamine and phosphocholine, plays a functional role in the initiation of calcification and that ablation of PHOSPHO1 and TNAP function prevents skeletal mineralization. *Phospho1*^*−/−*^ mice display growth plate abnormalities, spontaneous fractures, bowed long bones, osteomalacia, and scoliosis in early life. Primary cultures of *Phospho1*^*−/−*^ tibial growth plate chondrocytes and chondrocyte-derived matrix vesicles (MVs) show reduced mineralizing ability, and plasma samples from *Phospho1*^*−/−*^ mice show reduced levels of TNAP and elevated plasma PP_*i*_ concentrations. However, transgenic overexpression of TNAP does not correct the bone phenotype in *Phospho1*^*−/−*^ mice despite normalization of their plasma PP_*i*_ levels. In contrast, double ablation of PHOSPHO1 and TNAP function leads to the complete absence of skeletal mineralization and perinatal lethality. We conclude that PHOSPHO1 has a nonredundant functional role during endochondral ossification, and based on these data and a review of the current literature, we propose an inclusive model of skeletal calcification that involves intravesicular PHOSPHO1 function and P_*i*_ influx into MVs in the initiation of mineralization and the functions of TNAP, nucleotide pyrophosphatase phosphodiesterase-1, and collagen in the extravesicular progression of mineralization. © 2011 American Society for Bone and Mineral Research.

## Introduction

In the process of endochondral bone formation, chondrocytes and osteoblasts mineralize their extracellular matrix (ECM) at least in part by promoting deposition of crystalline hydroxyapatite (HA) in the sheltered interior of membrane-bounded matrix vesicles (MVs)—submicroscopic extracellular membrane–invested bodies enriched in phosphatases.(1,2) Early mineralization takes place inside these organelles, which serve as a site for Ca^2+^ and P_*i*_ accumulation to initiate the deposition of HA crystals.(3,4) In a second step, MV membranes subsequently rupture and/or break down, exposing preformed HA to the extracellular fluid and allowing for propagation of HA deposition within the ECM. Inorganic pyrophosphate (PP_*i*_) suppresses HA crystal formation and propagation and acts as a potent calcification inhibitor in biologic fluids.(5) Three molecules have been identified as central regulators of extracellular PP_*i*_ levels, namely, tissue-nonspecific alkaline phosphatase (TNAP), which is the primary enzyme that hydrolyzes PP_*i*_ in the ECM(6–10); nucleotide pyrophosphatase phosphodiesterase 1 (NPP1), which generates PP_*i*_ ectoplasmically from nucleoside triphosphates(11,12); and the multiple-pass transmembrane protein ANK, which mediates intracellular to extracellular channeling of PP_*i*_.(13,14)

TNAP is expressed at high levels in skeletal tissues, where it is found on the cell surfaces of odontoblasts, chondrocytes, and osteoblasts, including the membranes of their shed MVs.(15) Accumulation of PP_*i*_ in skeletal tissue caused by loss of TNAP's pyrophosphatase function leads to hypophosphatasia (HPP), an inborn error of metabolism characterized by rickets and osteomalacia.(16,17) Mice deficient in TNAP function (*Akp2*^*−/−*^) phenocopy infantile HPP; that is, they are born with normally calcified skeletons but by postnatal days 6 to 10, hypomineralization of the skeleton becomes apparent and worsens with age until their early demise by postnatal day 20.(18,19) The failure of bones to calcify after birth appears to result from a block in the propagation of HA in the ECM beyond the confines of the MV membrane(20,21) as a consequence of accumulated levels of PP_*i*_ in the ECM resulting from the lack of TNAP's pyrophosphatase function(9,10,17,22) together with the concomitant pyrophosphate-induced increase in osteoblast production of osteopontin, another potent inhibitor of calcification.(23,24) However, chondrocyte- and osteoblast-derived MVs in both HPP patients and *Akp2*^*−/−*^ mice retain the ability to initiate intravesicular mineral formation and contain HA crystals,(20,21) demonstrating that TNAP is not essential for the initiation of MV-mediated ECM mineralization and suggesting that other phosphatases or another mechanism might be responsible for this first step.

The phosphatase PHOSPHO1, first identified in the chick(25) as a member of the haloacid dehalogenase (HAD) superfamily of Mg^2+^-dependent hydrolases,(26) is expressed at levels 100-fold higher in mineralizing than in nonmineralizing tissues.(27) PHOSPHO1 shows high phosphohydrolase activity toward phosphoethanolamine (PEA) and phosphocholine (PCho),(28) is present and active inside chondrocyte- and osteoblast-derived MVs,(29) and the use of small-molecule compounds to inhibit PHOSPHO1 activity in *Akp2*^*−/−*^ MVs led to a significant decrease in MV-mediated calcification in vitro.(30) We surmised that PHOSPHO1 is involved in the first step of MV-mediated initiation of mineralization during endochondral ossification. In this article, we demonstrate conclusively the functional role of PHOSPHO1 during endochondral ossification and provide a unified, comprehensive model of the mechanisms of initiation of skeletal mineralization.

## Methods

### Mice

*Phospho1-R74X* null mutant (*Phospho1*^*−/−*^) mice were generated by *N*-ethyl-*N*-nitrosourea mutagenesis (ENU) in the C3HeB/FeJ (Stock No. 000658, Jackson Laboratories, Bar Harbor, ME, USA) background and bred to C57Bl/6 mice to segregate other possible undesired mutations. The generation of *Akp2*^*−/−*^ mice has been reported previously.(18) The *Akp2*^*−/−*^ mice used in this study were hybrids of C57Bl/6X129J mouse strains. The generation and characterization of the *ApoE-Tnap* transgenic mouse line has been described previously.(10) The homozygote mice exhibit up to 50-fold higher plasma levels of TNAP, produced primarily by the liver. The respective Institutional Animal Care and Use Committees (IACUCs) approved all animal studies.

### Tissue analysis

Whole-mount skeletal preparations were processed as before.(9,22) The lumbar spines, tibias, and femurs of 10-day-old mice and whole bodies of E16.5 embryos were fixed in PBS containing 4% (vol/vol) paraformaldehyde or a fixative containing 4% paraformaldehyde and 1% glutaraldehyde solution in 0.1 M sodium cacodylate buffer, pH. 7.2. Optimal cutting temperature compound (OCT) or paraffin sections were stained with the hematoxylin and eosin, alizarin red/alcian blue, von Kossa/van Gieson, and von Kossa/toluidine blue stains using standard procedures.(10,32,33) Von Kossa/van Gieson–stained slides were used for quantification of osteoid volume using the Bioquant Osteo Software (Bioquant Osteoanalysis Co., Nashville, TN, USA). Whole-body radiographic images were taken using an MX20 Specimen Radiograph System (Faxitron X-ray Corporation, Chicago, IL, USA) at different developmental ages (days 1, 3, and 10, 1 month, and 1 year). Tibia and femur lengths were measured using calipers. Micro–computed tomographic (µCT) analysis was carried out as described before.(21,23,31) Protein extracts (100 µg) from long bones of the *Phospho1*^*−/−*^ and WT mice were obtained as described previously(31) and used for Western blotting. PHOSPHO1 protein was detected with a recombinant human Fab antibody fragment selected against a human recombinant PHOSPHO1 (AbD05643.1) at a concentration of 1 µg/mL (AbD Serotec, MorphosysAG, Martinsried/Planegg, Germany). Recombinant human PHOSPHO1 protein(30) (20 ng) was used as a positive control.

### Cell-based assays

Primary calvarial osteoblasts were isolated from 1- to 3-day-old pups, and primary chondrocytes were isolated from the knee joint growth plates of 5-day-old pups by collagenase digestion, as described previously.(9,22,23) RNA was extracted using RNAeasy Pus Kit (Qiagen, Valencia, CA, USA). Specific RNA transcripts (mRNA) were quantified by real-time PCR using dual-labeled hydrolysis probes (FAM-TAMRA) (see Supplemental Text). Alizarin red S binding assay was performed using a standard method.(34) MVs were isolated from primary osteoblasts and chondrocytes by collagenase digestion and assayed for their calcification ability, as described previously.(35)

### Biochemical assays

Blood was collected by cardiac puncture or by eye bleed into lithium heparin tubes. TNAP and NPP1 activities were measured using colorimetric assays, as described previously.(35) PP_*i*_ concentrations were measured as described previously.(34)

### Statistical analysis

All measurements were performed at least in triplicate. Results are expressed as mean ± SEM and mean ± SD for µCT analysis of trabecular and cortical bone and ashing analysis. The data were analyzed using Student's *t* test. For µCT analysis, a Mann-Whitney test was conducted instead of a *t* test. For analysis of the mineral content, a rank-sum test was used. *P* values less than .05 are considered significant.

## Results

### *Phospho1*^*−/−*^ mice exhibit poor weight gain, growth plate and skeletal abnormalities, and thoracic scoliosis

**Fig. 1:**
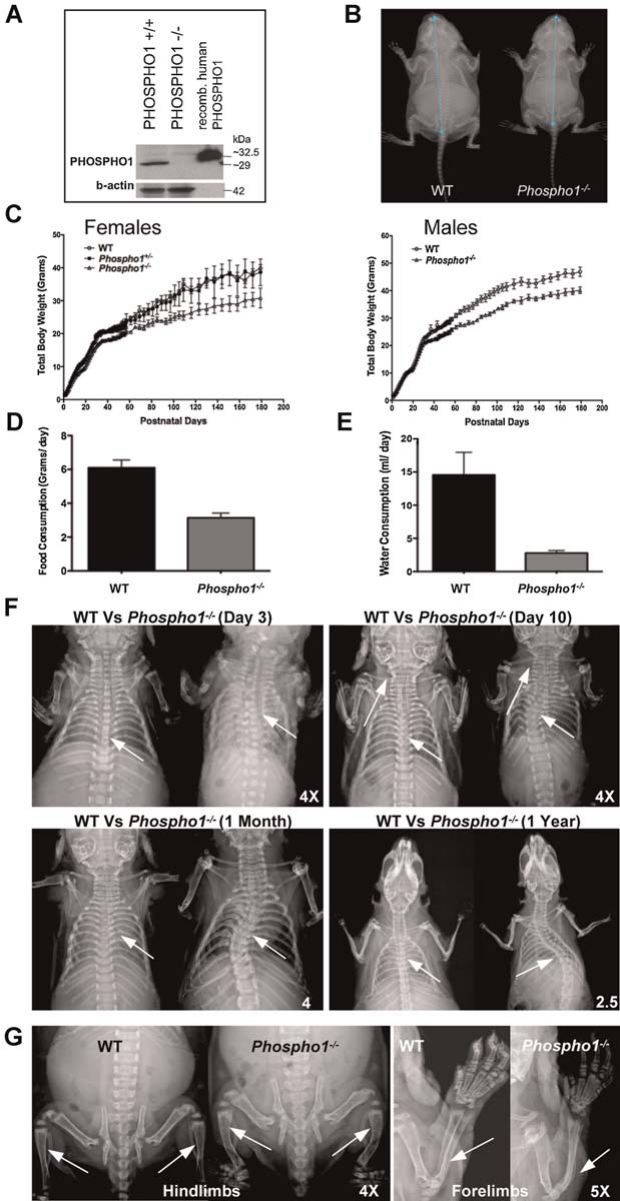
Phenotypic abnormalities in *Phospho1-R74X* (*Phospho1^−/−^*) mice. (*A*) Western blot showing the absence of PHOSPHO1 protein in *Phospho1^−/−^* mice. (*B*) Radiographic images of 10-day-old male mice showing the smaller size of *Phospho1^−/−^* compared with WT mice. (*C*) Body weights of WT, *Phospho1^+/−^*, and *Phospho1^−/−^* female mice and *Phospho1^−/−^* male mice from birth onwards. (*D*) Food (*N* = 4, *p* = .0001) and (*E*) water (*N* = 4, *p* = .0024) consumption by WT and *Phospho1^−/−^* mice measured per day for 3 consecutive days. (*F*) Radiographic images of WT and *Phospho1^−/−^* mice at 3 and 10 days of life, 1 month, and 1 year of age. *Phospho1^−/−^* mice showed clavicle (*arrow*) and rib deformities and scoliosis (*arrow*), clearly evident at 1 month of age in all *Phospho1^−/−^* mice, which becomes progressively worse with age. (*G*) Bowed long bones of both the hind and forelimbs, and evidence of spontaneous greenstick fractures is apparent.

*Phospho1-R74X* null mutant (*Phospho1*^*−/−*^) mice were generated by ENU mutagenesis. The absence of PHOSPHO1 protein in these mice was confirmed by Western blot analysis (Fig. 1*A*). The protein band of approximately 29 kDa corresponding to the native PHOSPHO1 protein can be seen in protein extracts of long bones of WT mice but not in the *Phospho1*^*−/−*^ samples.

**Fig. 2:**
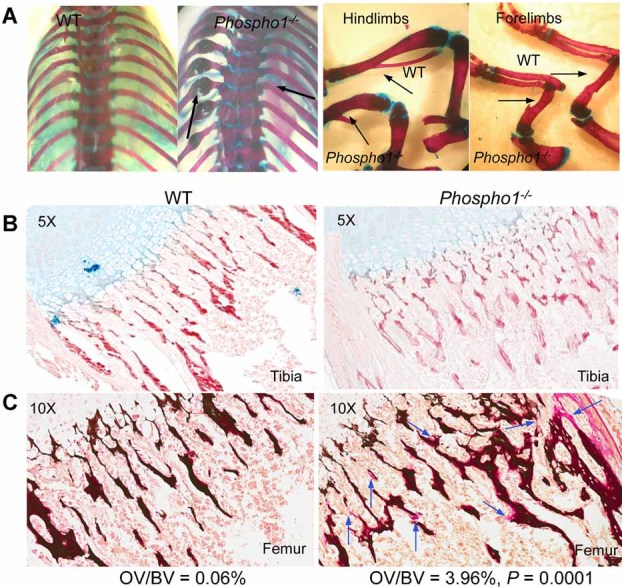
Whole mounts and histologic analyses of tibias and femurs of 10-day-old WT and *Phospho1^−/−^* mice. (*A*) Alizarin red/alcian blue staining of the whole skeleton of WT and *Phospho1^−/−^* mice. *Phospho1^−/−^* mice show callus formation at the sites of fractures in the ribs (*arrows*) and curved long bones in both hind and forelimbs (*arrows*). Tibial sections at the knee joint show growth plates of the WT and *Phospho1^−/−^* mice stained with (*B*) alizarin red/alcian blue and reveal reduced mineralization in *Phospho1^−/−^* mice. (*C*) Von Kossa/van Gieson staining of the femoral section at the knee joint reveals trabecular bone surrounded by widespread, extended osteoid in *Phospho1^−/−^* mice.

Both male and female *Phospho1*^*−/−*^ mice are smaller than age-matched heterozygous and WT controls (Fig. 1*B*) and exhibit growth retardation (Fig. 1*C*), where bones from 1-month-old male mice are shorter (ie, tibia: 16.3 ± 0.2 mm and 14.1 ± 0.6 mm, *p* = .0002; and femur: 12.4 ± 0.5 mm and 11.4 ± 0.6 mm, *p* = .0045, for WT and *Phospho1*^*−/−*^ mice, respectively). The difference in body weights is more prominent after 2 months of age and remains persistent thereafter. Studies using metabolic cages revealed that *Phospho1*^*−/−*^ mice eat (Fig. 1*D*) and drink (Fig. 1*E*) considerably less than WT littermates. Visual observation of the food pellets revealed less evidence of chewing in the *Phospho1*^*−/−*^ mice. Thoracic scoliosis was present in approximately 30% to 40% of the *Phospho1*^*−/−*^ mice on day 10, but at 1 month of age, scoliosis was clearly evident in 100% of *Phospho1*^*−/−*^ mice, and this spine deformity worsened progressively and became very prominent at 1 year of age (Fig. 1*F*). Greenstick fractures were present from postnatal day 1 in the vertebrae and hind and forelimbs of *Phospho1*^*−/−*^ mice (Fig. 1*G*). Whole-mount skeletal preparations of 10-day-old mice showed callus formation at the sites of fractures in the ribs of *Phospho1*^*−/−*^ mice and curved long bones in the hind and forelimbs (Fig. 2*A*).

**Fig. 3:**
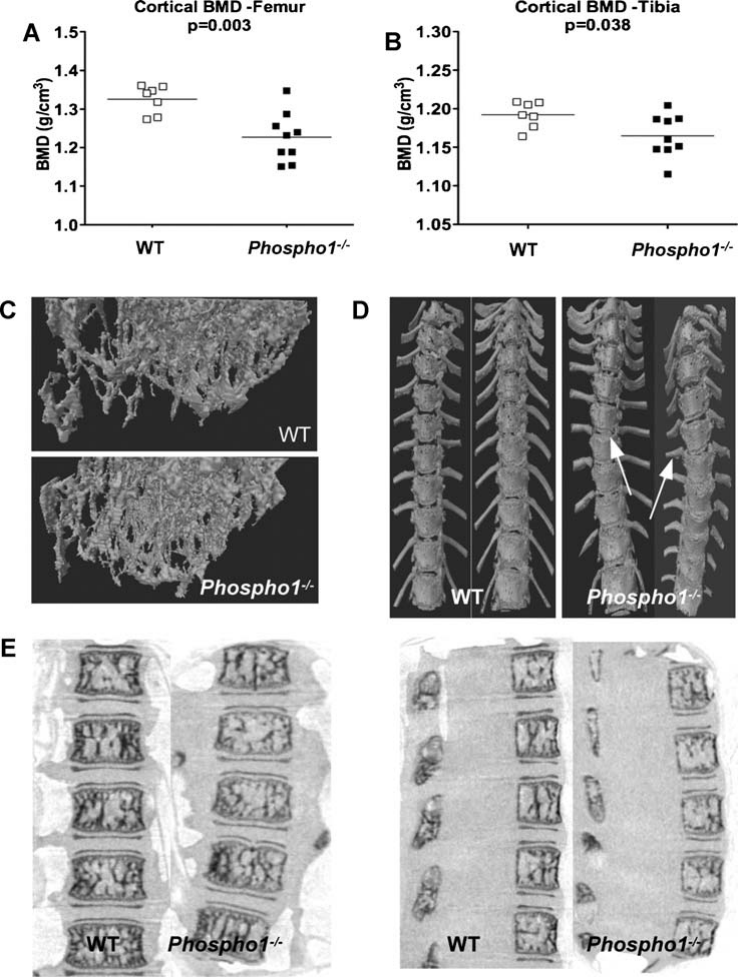
µCT analysis of 1-month-old WT and *Phospho1^−/−^* mice. (*A*, *B*) Decreased cortical BMD in femur (*p* = .003) and tibia (*p* = .038) of 1-month-old male *Phospho1^−/−^* mice (WT, *N* = 7; *Phospho1^−/−^* mice, *N* = 9). (*C*) µCT images showing increased trabeculi and decreased trabecular spaces in tibia of the *Phospho1^−/−^* bones. (*D*) µCT images of the spine show scoliosis in *Phospho1^−/−^* mice. (*E*) High-resolution µCT images of the spine showing no obvious morphologic abnormalities in the individual *Phospho1^−/−^* vertebrae.

Histologic analysis of the cryosections of tibia stained with alizarin red/alcian blue staining (Fig. 2*B*) showed reduced mineralization of the trabecular bone. About 10% to 15% of 10-day-old *Phospho1*^*−/−*^ mice showed complete absence of secondary ossification centers. Von Kossa/van Gieson staining (Fig. 2*C*) revealed characteristics of osteomalacia in *Phospho1*^*−/−*^ mice: widespread excessive osteoid (OV/BV = 3.96% in *Phospho1*^*−/−*^ mice versus 0.06% in WT mice, *p* = .0001) and increased width of osteoid at the surfaces of both trabecular and cortical bone. Histochemical staining showed markedly reduced levels of TNAP activity in the hypertrophic chondrocytes, metaphyseal trabecular bone, and secondary ossification centers in the femur of *Phospho1*^*−/−*^ mice (data not shown). µCT analysis of 1-month-old *Phospho1*^*−/−*^ and WT mice showed no significant difference in tibia and femur trabecular BV/TV ratio (Supplemental Table S1), but the cortical bone mineral density (BMD) was significantly reduced in both femur (*p* = .003) and tibia (*p* = .038; Fig. 3*A, B*). Ashing analysis of the humeri confirmed the decreased BMD of the *Phospho1*^*−/−*^ mice, where the percent ash content of the WT mice was significantly higher than the percent ash content of the *Phospho1*^*−/−*^ mice (55% ± 4% versus 50% ± 7%, *p* = 0.038). We detected increased cortical porosity in the femur and decreased cortical thickness in the tibia of *Phospho1*^*−/−*^ mice (Supplemental Table S1). The increased BMD measured in the medular cavity of the tibia is likely a result of the increased trabecular number and decreased spacing noted in the *Phospho1*^*−/−*^ mice (Fig. 3*C* and Supplemental Table S1). µCT images of the spine, taken at 1 month of age, showed scoliosis in *Phospho1*^*−/−*^ mice (Fig. 3*D*). Both dextro- and levoscoliosis was observed in these mice, but high-resolution µCT images did not reveal any morphologic vertebral abnormalities (Fig. 3*E*).

### Biochemical changes in *Phospho1*^*−/−*^ mice

Serum glucose, blood urea nitrogen, creatinine, albumin, globulin, total protein, total bilirubin, sodium, potassium, and calcium were normal in 10-day-old as well as 1-year-old *Phospho1*^*−/−*^ mice (Supplemental Table S2). A slight hyperphosphatemia was observed transiently in 10-day-old *Phospho1*^*−/−*^ mice. A comprehensive pathologic exam of soft tissues did not reveal any abnormalities in the kidneys, liver, spleen, lungs, heart, thymus, or gastrointestinal tract of 20-day-old *Phospho1*^*−/−*^ mice. We measured expression of *Runx2*, *Col2a1*, *Col10a1*, *aggrecan*, and *MMP1*3 in 1-, 7-, and 14-day-old growth plate chondrocyte cultures from WT and *Phospho1*^*−/−*^ mice. We found a statistically significant decrease in *Col2a1*, *aggrecan*, and *MPP13* expression in *Phospho1*^*−/−*^ chondrocyte cultures and a statistically significant decrease in *Col10a1* expression in 14-day cultures (Supplemental Fig. S1).

**Fig. 4:**
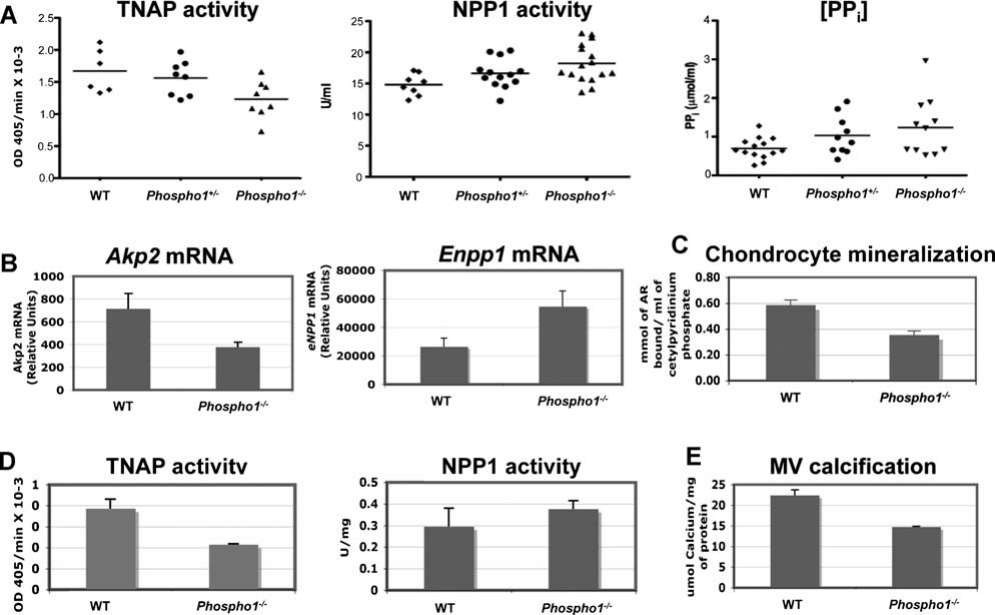
Biochemical and gene expression changes in *Phospho1^−/−^* mice. (*A*) One-year-old *Phospho1^−/−^* mice show reduced plasma TNAP activity [*N* = 6 (WT); *N* = 8 (*Phospho1^+/−^*); *N* = 8 (*Phospho1^−/−^*), *p* = .012], increased plasma NPP1 activity [*N* = 8 (WT); *N* = 13 (*Phospho1^+/−^*); *N* = 16 (*Phospho1^−/−^*), *p* = .004], and high plasma PP_*i*_ levels [*N* = 13 (WT); *N* = 10 (*Phospho1^+/−^*); *N* = 11 (*Phospho1^−/−^*), *p* = .009] compared with heterozygous and WT littermates. (*B*) Decreased *Akp2* and increased *Enpp1* mRNA expression in day 1 chondrocytes assessed by qPCR. Data are represented as mean ± SEM, *N* =3, experiments done in triplicates. (*C*) Decreased mineralization (alizarin red staining) in *Phospho1^−/−^* chondrocytes grown in the presence of mineralization medium containing ascorbic acid and β-glycerophosphate for 14 days and alizarin red measurements. (*D*) MVs from *Phospho1^−/−^* mice showed reduced TNAP activity (*p* = .016), increased NPP1 activity (*p* = .05), and (*E*) reduced calcification ability (*p* = .002) compared with WT MVs. Data are represented as mean ± SEM, *N* =3 experiments done in triplicate.

In agreement with the observation of the reduced levels of TNAP activity in the growth plates, metaphyseal trabecular bone and secondary ossification centers in *Phospho1*^*−/−*^ mice, we also found reduced levels of TNAP activity in the plasma of 1-year-old *Phospho1*^*−/−*^ mice (Fig. 4*A*; *p* = .012). We also observed increased plasma activity of NPP1 (*p* = .004), and as a consequence of the reduced TNAP and enhanced NPP1 activity, *Phospho1*^*−/−*^ mice had higher than normal levels of plasma PP_*i*_ (1.24 ± 0.2 µmol/L) compared with WT mice (0.7 ± 0.07 µmol/L, *p* = 0.009; Fig. 4*A*). These biochemical changes were confirmed at the mRNA level in cultures of both primary chondrocytes (Fig. 4*B*) and osteoblasts. WT chondrocytes were grown in culture for 14 days in the presence of differentiation medium containing ascorbic acid, and *Phospho1* mRNA expression was assessed on each day. The highest *Phospho1* gene expression was observed on day 1 of culture, and therefore, 1-day-old chondrocytes were used for TNAP (*Akp2*) and NPP1 (*Enpp1*) expression studies. In agreement with the biochemical measurements, quantitative PCR (qPCR) studies of mRNA isolated from 1-day-old chondrocytes cultures (Fig. 4*B*) revealed a 2-fold decrease in *Akp2* mRNA (*p* = .017), a 2.5-fold increase in *Enpp1* mRNA (*p* = 0.032), and a 1.6-fold increase in *Ank* mRNA (*p* = 0.038) in *Phospho1*^*−/−*^ cells compared with WT cells. The mineralizing ability of *Phospho1*^*−/−*^ primary chondrocytes was reduced in comparison with WT chondrocytes (0.59 ± 0.04 versus 0.36 ± 0.03 mmol of alizarin red–bound/cetyl pyridinium phosphate in WT and *Phospho1*^*−/−*^ mice, respectively, *p* = 0.009; Fig. 4*C*). These observations were extended to the level of the chondrocyte-derived MVs, where again we observed a decrease in TNAP activity (Fig. 4*D*; *p* = .016) and an increase in NPP1 activity (Fig. 4*D*; *p* = .05) in MVs isolated from *Phospho1*^*−/−*^ mice compared with WT mice. The *Phospho1*^*−/−*^ MVs showed reduced calcification ability (∼14.76 µmol calcium/mg of protein) compared with WT MVs (∼22.44 µmol calcium/mg of protein; Fig. 4*E*; *p* = .002).

### The *Phospho1*^*−/−*^ phenotype is not rescued by overexpression of TNAP

**Fig. 5:**
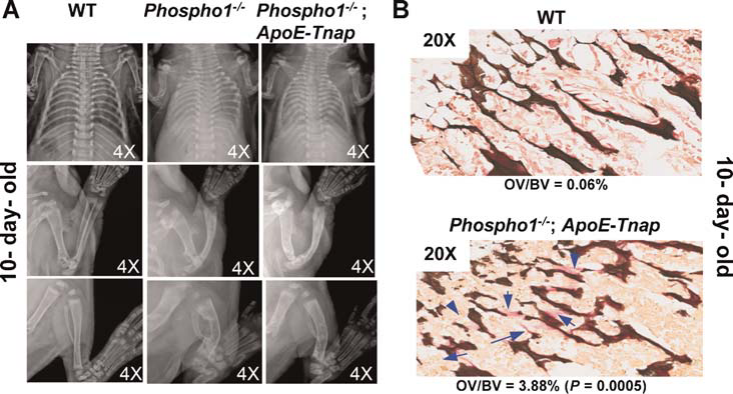
The *Phospho1^−/−^* phenotype is not rescued by overexpression of TNAP; X-ray images of the skeleton of a 10-day-old [*Phospho1^−/−^*; *ApoE-Tnap*] mice. No improvement in the skeletal phenotype of 10-day-old *Phospho1^−/−^* mice was observed by overexpressing TNAP as assessed by (*A*) radiography and (*B*) osteoid measurement in the femur after von Kossa/van Gieson staining.

The increased levels of plasma PP_*i*_ and the reduced activity of TNAP observed in *Phospho1*^*−/−*^ mice are reminiscent of the changes observed in HPP, where the elevated PP_*i*_ levels are responsible for the ensuing rickets and osteomalacia characteristic of this disease.(9,16,17,19) The HPP phenotype can be completely rescued by cross-breeding *Tnap* null (*Akp2*^*−/−*^) mice with transgenic mice overexpressing TNAP under control of the *ApoE* promoter.(10) In order to assess whether the phenotypic abnormalities observed in the *Phospho1*^*−/−*^ mice were attributable to altered PP_*i*_ metabolism, we cross-bred *Phospho1*^*−/−*^ to *ApoE-Tnap* transgenic mice. [*Phospho1*^*−/−*^*; ApoE-Tnap*] mice did not show any significant improvement in their skeletal phenotype at 10 days of age, as assessed by radiography and histology of the femur (Fig. 5*A, B*), despite a significant reduction in the circulating levels of PP_*i*_ (*Phospho1*^*−/−*^ mice = 1.02 ± 0.04 µmol/L and [*Phospho1*^*−/−*^*; ApoE-Tnap*] mice = 0.89 ± 0.05 µmol/L, *p* = .0348) and a significant increase (∼4 fold) in the plasma levels of TNAP (*Phospho1*^*−/−*^ mice = 197 ± 25 U/L and [*Phospho1*^*−/−*^*; ApoE-Tnap*] mice = 933 ± 130 U/L, *p* < .0001). NPP1 levels did not show any significant change (*Phospho1*^*−/−*^ mice = 344 ± 19 U/L and [*Phospho1*^*−/−*^*; ApoE-Tnap*] mice = 307 ± 19 U/L, *p* = .26). Analysis of 3.5- and 7-month-old [*Phospho1*^*−/−*^*; ApoE-Tnap*] mice showed no correction of the skeletal phenotype despite the persistently high levels of plasma TNAP activity ([*Phospho1*^*−/−*^*; ApoE-Tnap*] mice = 1940 ± 242, *p* = .0003 U/L; *Phospho1*^*−/−*^ mice = 61 ± 20 U/L, and WT mice = 179 ± 18 U/L) and normal levels of PP_*i*_ ([*Phospho1*^*−/−*^*; ApoE-Tnap*] mice = 3.2 ± 0.4 µmol/L, *p* = .0018, *N* = 8; *Phospho1*^*−/−*^ mice = 4.4 ± 0.6 µmol/L, *N* = 5, and WT mice = 2.9 ± 0.1 µmol/L, *N* = 12). [*Phospho1*^*−/−*^*; ApoE-Tnap*] mice manifested the same decrease in food and water consumption observed in the *Phospho1*^*−/−*^ mice.

### Nonredundant role of PHOSPHO1 in skeletal mineralization

**Fig. 6:**
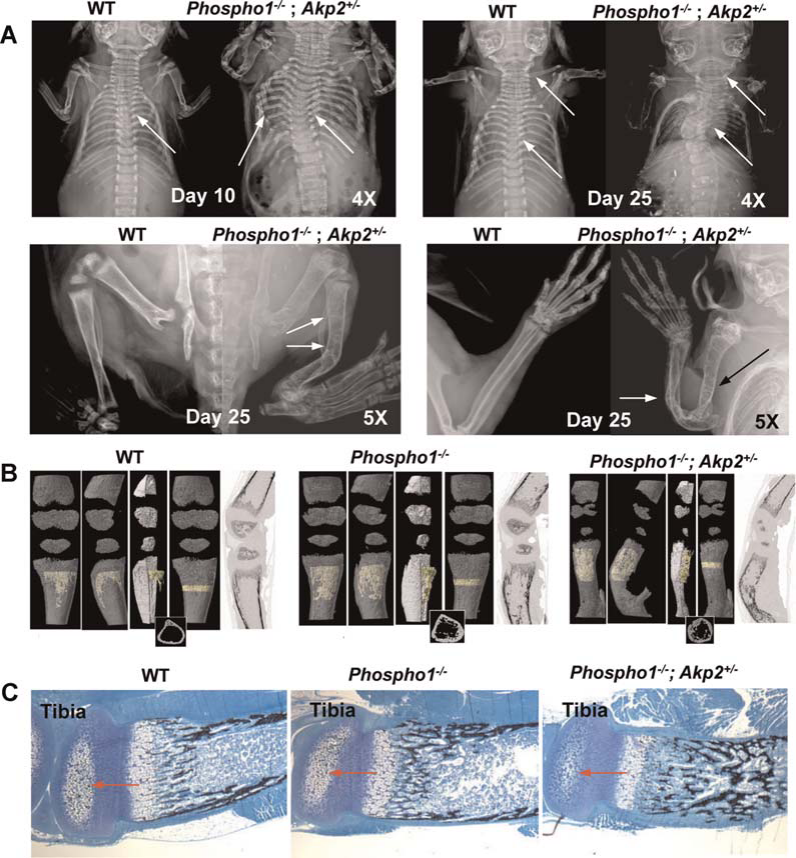
Worsening of the skeletal abnormalities on ablating *Akp2* alleles in *Phospho1^−/−^* mice. (*A*) Radiographic images of WT and [*Phospho1^−/−^*; *Akp2*^+/−^] mice on postnatal days 10 and 25. All the skeletal abnormalities of *Phospho1^−/−^* mice were aggravated in [*Phospho1^−/−^*; *Akp2^+/−^*] mice. Multiple fractures and highly deformed long bones were observed from day 1, and scoliosis was evident as early as day 10 in all the [*Phospho1^−/−^*; *Akp2^+/−^*] mice. (*B*) µCT images of WT, *Phospho1^−/−^*, and [*Phospho1^−/−^*; *Akp2^+/−^*] mice. Highly curved tibias and smaller secondary ossification centers in [*Phospho1^−/−^*; *Akp2^+/−^*] mice. (*C*) Sections (1 µm) stained with von Kossa/toluidine blue show deformed cortical bone and reduced secondary ossification centers in [*Phospho1^−/−^*; *Akp2^+/−^*] and *Phospho1^−/−^* mice.

The fact that overexpression of TNAP does not prevent the development of skeletal abnormalities in the *Phospho1*^*−/−*^ mice, despite correction of plasma PP_*i*_ and greatly elevated TNAP levels, suggests that PHOSPHO1 functions through a pathway that is distinct from that of TNAP. We predicted, therefore, that the simultaneous ablation of PHOSPHO1 and TNAP function would severely compound the mineralization phenotype characteristic of each individual knockout model. Deleting a single allele of *Akp2* (TNAP) in the *Phospho1* null background aggravated the skeletal phenotype of *Phospho1*^*−/−*^ mice (Fig. 6*A*). [*Phospho1*^*−/−*^*; Akp2*^*+/−*^] pups were born but at a greatly reduced rate (5.8% compared with the expected 12.5%; Supplemental Table S3). Multiple fractures were seen from postnatal day 1, and prominent scoliosis was observed already on postnatal day 10 in [*Phospho1*^*−/−*^*; Akp2*^*+/−*^] mice compared with 1 month of age in *Phospho1*^*−/−*^ mice. µCT analysis of the bones in [*Phospho1*^*−/−*^; *Akp2*^*+/−*^] mice also showed highly curved tibias at the site of the fractures, and secondary ossification centers were smaller and less developed than in the *Phospho1*^*−/−*^ mice (Fig. 6*B*). Histologic analysis of the von Kossa/toluidine blue–stained sections of the tibias (Fig. 6*C*) also showed reduced secondary ossification centers and deformed cortical bone compared with *Phospho1*^*−/−*^ and WT mice. Similar findings also were observed in the digits and third metatarsal. Transmission electron microscopic images of the third metatarsal bones showed decreased bone mineralization in the ECM in the *Phospho1*^*−/−*^ mice and greatly reduced bone ECM mineralization (osteoidosis) in the [*Phospho1*^*−/−*^*; Akp2*^*+/−*^] mice.

**Fig. 7:**
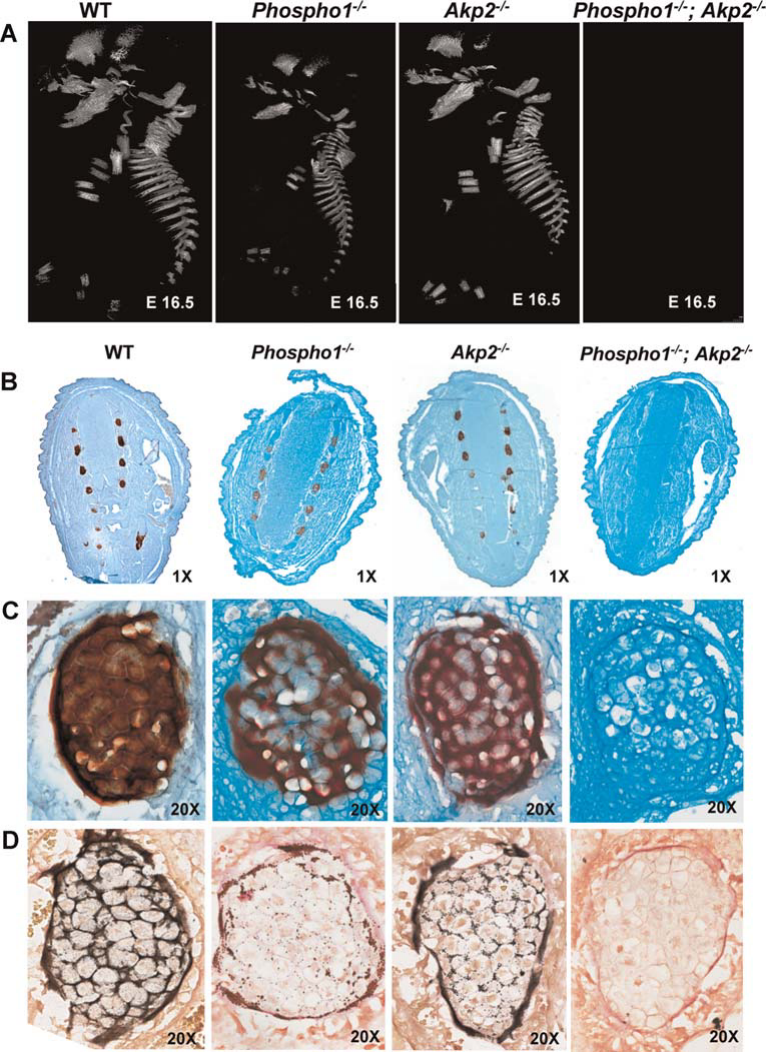
Lack of skeletal mineralization in [*Phospho1^−/−^*; *Akp2^−/−^*] double-knockout mice. (*A*) µCT image of an [*Phospho1^−/−^; Akp2^−/−^*] E16.5 embryo shows complete absence of skeletal mineralization compared with WT, *Phospho1^−/−^*, and *Akp2^−/−^* embryos. (*B*) Alizarin red/alcian blue staining of the transversal section from the lower body of E16.5 WT, *Phospho1^−/−^*, *Akp2^−/−^*, and [*Phospho1^−/−^*; *Akp2^−/−^*] double-knockout embryos. Higher magnification (×20) of a vertebral bone showing completely absent mineralization in the [*Phospho1^−/−^*; *Akp2^−/−^*] specimen and reduced mineralization in the *Phospho1^−/−^* embryo, as detected by (*C*) alizarin red/alcian blue staining and (*D*) von Kossa/van Gieson staining.

Of 272 pups born to *Phospho1*^*+/−*^ × *Akp2*^*+/−*^ and [*Phospho1*^*−/−*^; *Akp2*^*+/−*^] × [*Phospho1*^*−/−*^; *Akp2*^*+/−*^] matings (Supplemental Table S3), only one double-knockout [*Phospho1*^*−/−*^*; Akp2*^*−/−*^] pup was born, and that was a stillbirth. µCT analysis of the P_0_ stillborn [*Phospho1*^*−/−*^; *Akp2*^*−/−*^] specimen revealed complete lack of mineralization in the appendicular skeleton (Supplemental Fig. S2). The axial skeleton also was highly deformed and only partially mineralized, as were some craniofacial bones. Since ablating both PHOSPHO1 and TNAP function appeared perinatal lethal, we examined [*Phospho1*^*−/−*^; *Akp2*^*−/−*^] embryos. The expected percentage and numbers of E16.5 [*Phospho1*^*−/−*^; *Akp2*^*−/−*^] double-knockout embryos were obtained and studied (*N* = 6). Figure 7*A* shows the µCT analysis of an E16.5 [*Phospho1*^*−/−*^*; Akp2*^*−/−*^] embryo showing complete lack of skeletal mineralization. Alizarin red/Alcian blue staining of the transversal sections of the embryos shows reduced calcification of the vertebral bones and femur of the E16.5 *Phospho1*^*−/−*^ embryo compared to the WT E16.5 control and *Akp2*^*−/−*^ embryos. The [*Phospho1*^*−/−*^; *Akp2*^*−/−*^] embryos showed a complete absence of skeletal mineralization (bone and cartilage; Fig. 7*B, C*). Von Kossa and van Gieson staining of the vertebral bones also showed a complete lack of mineralization in the [*Phospho1*^*−/−*^; *Akp2*^*−/−*^] specimens (Fig. 7*D*).

## Discussion

We have studied the role of PHOSPHO1 during endochondral ossification by examining the phenotypic alterations resulting from ablation of PHOSPHO1 function alone or in combination with TNAP deficiency. Lack of PHOSPHO1 caused a decrease in growth rate, endochondral growth plate, and skeletal abnormalities that included decrease or loss of secondary ossification centers, decreased bone mineral density, spontaneous fractures, osteomalacia, and scoliosis. Cultured growth plate chondrocytes showed decreased expression of differentiation markers, including *Col2a1*, *aggrecan*, *MMP13*, and *Col10a1*, indicative of a cellular growth plate phenotype, and chondrocytes and osteoblasts from *Phospho1*^*−/−*^ mice, as well as their derived MVs, displayed a reduced ability to calcify, consistent with the reduced mineralization of their skeleton, clearly demonstrating that PHOSPHO1 is required for normal endochondral ossification.

Metabolic studies indicated that the reduced growth rate of *Phospho1*^*−/−*^ mice was attributable to reduced food and water consumption. Visual observation of the food pellets revealed less evidence of chewing in the *Phospho1*^*−/−*^ mice. We surmise that this might be caused by softer jaws and/or teeth and reduced mobility resulting from the hypomineralization phenotype in the *Phospho1*^*−/−*^ mice. We are currently examining tooth development and tooth mineralization to better understand this aspect of the phenotype in *Phospho1*^*−/−*^ mice. Functional adaptation and changes in mechanical loading(36,37) can explain the different architectural changes noted in the *Phospho1*^*−/−*^ mice in the tibia and femur. Both levo- and dextroscoliosis can be seen in *Phospho1*^*−/−*^ mice, but detailed examination of the vertebrae by µCT ruled out the presence of obvious morphologic vertebral abnormalities (hemivertebrae or fused vertebrae), indicating that the scoliosis, as well as the bowing of long bones, is likely caused by muscular forces acting on the malleable hypomineralized matrix of the *Phospho1*^*−/−*^ mice.(38,39)

Cultures of *Phospho1*^*−/−*^ growth plate chondrocytes revealed decreased expression of *Col2a1*, *aggrecan*, *MMP13*, and *Col10a1*, indicating a cell differentiation phenotype compatible with the subtle morphologic changes observed in the histologic sections of the growth plates. Of particular interest was the fact that the *Phospho1*^*−/−*^ mice showed enhanced production (high NPP1 activity), enhanced transport (high ANK expression), and decreased degradation (reduced TNAP activity) of PP_*i*_, a situation that was highly reminiscent of that encountered in *Akp2*^*−/−*^ mice (deficient in TNAP), where elevated levels of PP_*i*_ explained the rickets/osteomalacia characteristic of HPP in this knockout model.(19) However, correcting PP_*i*_ levels in *Akp2*^*−/−*^ mice, either via transgenic overexpression of TNAP into the *Akp2* null background, that is, in [*Akp2*^*−/−*^*; ApoE-Tnap*] mice,(10) or via the use of enzyme-replacement therapy with a bone-targeted form of TNAP,(32) completely prevented the development of skeletal and dental abnormalities characteristic of this model of infantile HPP. This was not the case, however, when PP_*i*_ levels were reduced and plasma TNAP levels were highly increased in *Phospho1*^*−/−*^ mice by cross-breeding them with the same *ApoE-Tnap* transgenic mice. These data indicate that while PHOSPHO1 function can influence expression of the molecules involved in PP_*i*_ metabolism, that is, NPP1, ANK, and TNAP, the phenotypic abnormalities in *Phospho1*^*−/−*^ mice cannot be explained simply by the resulting modulations in PP_*i*_ concentrations. Furthermore, the fact that the double ablation of PHOSPHO1 and TNAP function leads to an essentially complete absence of mineralization provides compelling experimental evidence supporting the assertion that PHOSPHO1 and TNAP have independent, nonredundant roles during endonchondral ossification.

PHOSPHO1 is a soluble cytosolic enzyme that has specificity for phosphoethanolamine (PEA) and phosphocholine (PCho).(28) Both PEA and PCho are the two most abundant phosphomonoesters in cartilage,(40) and the PEA and PCho composition of the MV membrane decreases during mineralization, in conjunction with phospholipase C activity.(41) The low PCho accumulation in mineralizing compared with nonmineralizing cells is compatible with the upregulation of PHOSPHO1 activity in mineralizing cells, whose function reduces the levels of PEA and PCho in chondrocytes and osteoblasts.(42) The very low *K*_*m*_ values for both PEA and PCho (µM range) indicate that under physiologic conditions, PHOSPHO1 rapidly hydrolyzes both molecules.(28) Thus, through the enzymatic action of PHOSPHO1, as part of the mineralization process, P_*i*_ appears to be scavenged from PEA and PCho in order to generate the P_*i*_ concentration needed to establish a P_*i*_/PP_*i*_ ratio permissive for the initial formation of HA crystal inside the MVs. It is clear from our data that modulating PHOSPHO1 function influences the expression of the *Enpp1, Ank*, and *Akp2* genes, given the expression changes observed in the *Phospho1*^*−/−*^ cells. But why did the functional ablation of PHOSPHO1 not abolish initiation of HA deposition inside MVs, whereas there was a tremendous decrease in mineralization of the skeleton after the double ablation of PHOSPHO1 and TNAP? To answer this question, one must first review the literature regarding the expression of TNAP and NPP1 in the appendicular and axial skeletons and also review the kinetic properties of TNAP and NPP1 toward physiologic substrates ATP, ADP, and PP_*i*_ at the level of MVs.

It has been proposed that the role of TNAP in the bone matrix is to generate the P_*i*_ needed for HA crystallization.(7,43,44) However, TNAP also has been shown to hydrolyze the mineralization inhibitor PP_*i*_ to facilitate mineral precipitation and growth.(6,7) Previous studies from this laboratory and collaborators have shown conclusively that a major function of TNAP in bone tissue consists of hydrolyzing PP_*i*_ to maintain a proper concentration of this mineralization inhibitor to ensure normal bone mineralization,(8,9,21,22) and we have even shown that the coexpression of TNAP and fibrillar collagens is “necessary and sufficient” to cause ECM calcification.(10) The major conclusion of that article was that the pervasive presence of the potent calcification inhibitor PP_*i*_ in all body fluids prevents unwanted calcification in tissues other than bones and teeth. However, transgenic overexpression of TNAP can lead to a decrease in local PP_*i*_ concentrations that enable the spontaneous crystallization of ionic calcium and P_*i*_ to form bone mineral within a fibrillar/collagenous scaffold. In turn, lack of TNAP in *Akp2*^*−/−*^ mice leads to accumulation of extracellular PP_*i*_ that causes the rickets and dental abnormalities characteristic of infantile HPP.(19,32) Cross-breeding of *Akp2*^*−/−*^ to *Enpp1*^*−/−*^ mice leads to normalization of the extracellular PP_*i*_ levels and correction of the skeletal defects in [*Akp2*^*−/−*^*; Enpp1*^*−/−*^] double-knockout mice.(9) However, this genetic correction is only partial, with major improvements observed in the axial skeleton but only partial changes observed in the appendicular skeleton.(31) This is attributable to the different levels of expression of NPP1 in these skeletal environments; that is, NPP1 is highly expressed in the calvaria, but expression is much lower in the femurs/tibias of mice.(31) Thus ablation of the PP_*i*_-generating activity of NPP1 in the axial skeleton (calvarium and spine) of *Akp2*^*−/−*^ mice led to a significant reduction in PP_*i*_ production in skeletal sites that was sufficient to normalize PP_*i*_ concentrations and prevent hypomineralization. However, ablating the lower levels of NPP1 in the appendicular skeleton was not sufficient to adequately reverse PP_*i*_ levels back to normal in those sites, and inadequate mineralization persisted.(31) Recent data from our laboratory indicate that besides the PP_*i*_-generating activity of NPP1 in chondrocytes and osteoblasts, at the level of MVs, NPP1 can act as an efficient phosphatase, producing P_*i*_ from ATP, ADP, and PP_*i*_, but that this activity is evident only in the absence of TNAP, which is a much more efficient phosphatase for all these three physiologic substrates.(35) These new data help to explain why *Akp2*^*−/−*^ mice, which are null for TNAP activity, display an HPP phenotype that is less severe than the most severe cases of human HPP reported, such as lethal and perinatal HPP.(16,17) In the absence of TNAP, NPP1 can act as a backup pyrophosphatase in the extravesicular space to temporarily restrict the concentrations of extracellular PP_*i*_ to allow *Akp2*^*−/−*^ mice to develop normal mineralization for the first 6 days of life. After that, the hypomineralization abnormalities become apparent. This partial compensatory pyrophosphatase activity of NPP1 also explains why in the single stillborn [*Phospho1*^*−/−*^*; Akp2*^*−/−*^] double-knockout pup reported in this article, there was some partial mineralization of the axial skeleton.

Several articles have involved the action of ATPases in the initiation of endochondral ossification.(45–47) The article by Ciancaglini and collaborators clearly has documented that the major ATPase of MVs is TNAP but that NPP1 can act as an ATPase in the absence of TNAP.(35) In contrast, PHOSPHO1 is a very inefficient phosphatase when confronted with ATP, ADP, or PP_*i*_.(35) These data are very relevant to understanding the roles of organic and inorganic phosphates in endochondral ossification and in explaining the complete ablation of skeletal mineralization in [*Phospho1*^*−/−*^*; Akp2*^*−/−*^] double-knockout embryos. Calcification, both intravesicular and extravesicular, is abolished in [*Phospho1*^*−/−*^*; Akp2*^*−/−*^] embryos despite the availability of systemic P_*i*_ in these mice. This argues that organic phosphates, such as ATP or ADP, might act as the major source of P_*i*_ that is required for the initiation of calcification. Chondrocytes, osteoblasts, and their derived MVs express and use phosphate transporters on their membrane for uptake of P_*i*_.(48,49) We must conclude that the mineralizing cells consider it efficient to invest the energy required to generate and export ATP to be used for the local generation of P_*i*_ in the immediate environment of MVs and for subsequent incorporation into MVs via P_*i*_ transporters. Thus, in the absence of both PHOSPHO1 and TNAP function, there is complete lack of skeletal mineralization because there is no P_*i*_ generation from substrates attributable to the absence of TNAP's ATPase activity, and the levels of ATPase provided by NPP1 in the embryonic skeleton are clearly insufficient to allow calcification to proceed. However, some calcification still was observed in the axial skeleton of the single stillborn [*Phospho1*^*−/−*^*; Akp2*^*−/−*^] double-knockout pup likely attributable to P_*i*_ generation via the ATPase action of NPP1 and also by the concomitant restriction of extracellular PP_*i*_ concentrations by the pyrophosphatase activity of NPP1. Thus this provides an explanation why complete ablation of PHOSPHO1 function leads only to a decrease in the calcification ability of MVs but not to a complete lack of calcification, as we showed previously using small-molecule inhibitors of PHOSPHO1 activity(30) and in this article by the gene-knockout approach. Deletion of PHOSPHO1 would suppress intravesicular generation of P_*i*_ but would leave extravesicular P_*i*_ generation via TNAP's ATPase activity and influx via P_*i*_ transporters unaffected.

Integrating these data, it is now possible to propose an inclusive model for the initiation of skeletal mineralization that unifies a number of concepts and functions that have been considered contradictory in the past. Our unified model starts with the MVs as the site of initiation of mineralization (Supplemental Fig. S3). HA crystals appear inside the MVs favored by P_*i*_ accumulation resulting from a dual mechanism, that is, PHOSPHO1-mediated intravesicular production and transporter-mediated influx of P_*i*_ produced extravesicularly primarily by TNAP's ATPase activity or, secondarily, in the absence of TNAP by NPP1's ATPase activity. Organophosphate compounds, and perhaps also PP_*i*_, are the source of P_*i*_ for this initial step of calcification. Then extravesicular calcification is supported mainly by TNAP's pyrophosphatase activity and, secondarily, by NPP1's pyrophosphatase activity (in the absence of TNAP) and is driven by the availability of P_*i*_ and the presence of a collagenous fibrilar scaffold and guided by other ECM mineral-binding proteins. This proposed model, compatible with all available experimental data, takes into account the roles of both organic and inorganic phosphates in skeletal calcification and unifies the roles of MV- and collagen-mediated calcification as two separate but linked steps during endochondral ossification.
