# Supplementary figures and images for: Loss of Skeletal Mineralization by the Simultaneous Ablation of PHOSPHO1 and Alkaline Phosphatase Function: A Unified Model of the Mechanisms of Initiation of Skeletal Calcification
Source: J Bone Miner Res. 2010 Aug 3;26(2):286–97. doi: 10.1002/jbmr.195 (PMC3179344; doi:10.1002/jbmr.195)

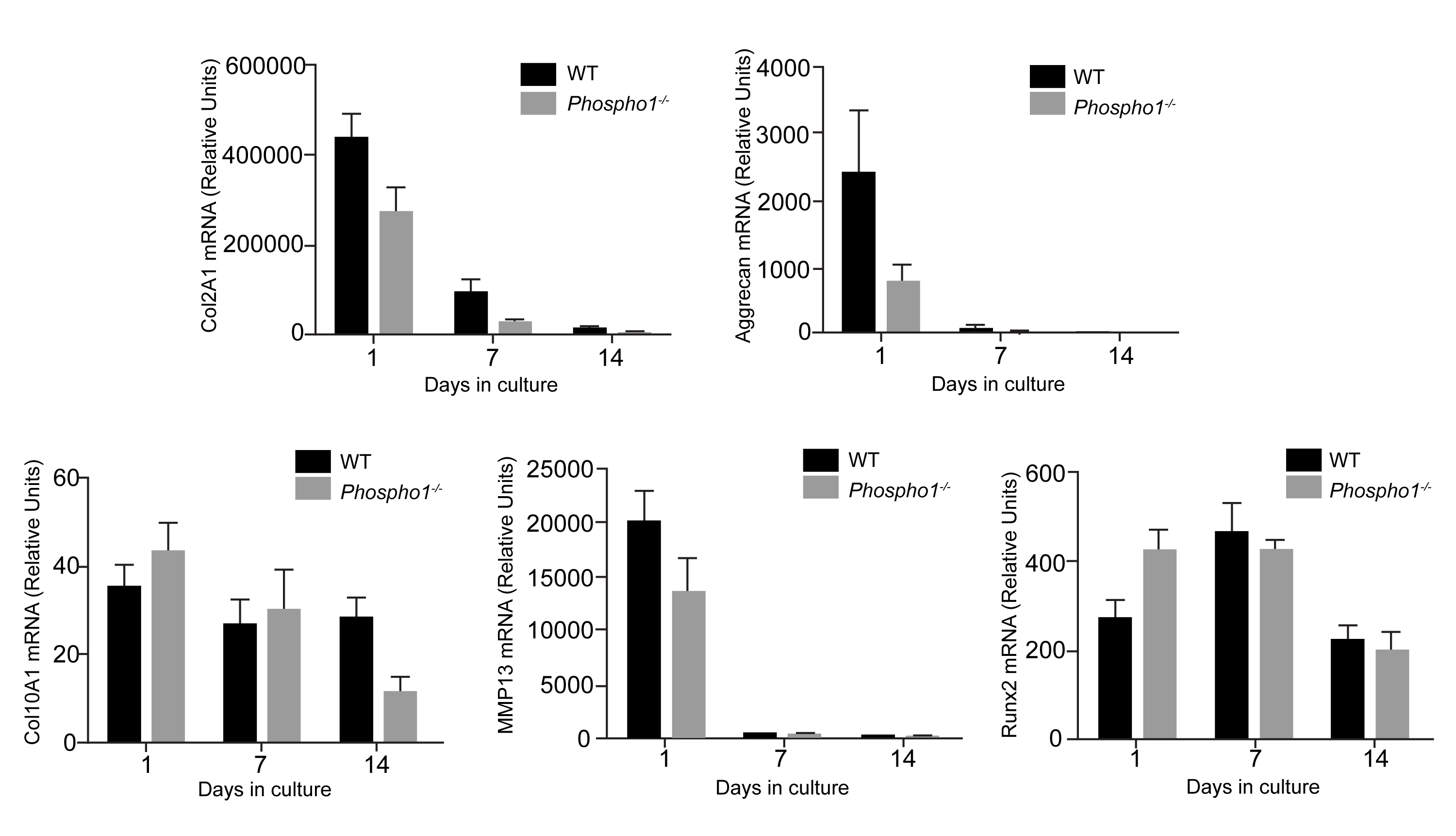

Supplement: Supplementary file 1 [file jbmr0026-0286-SD1.tif]

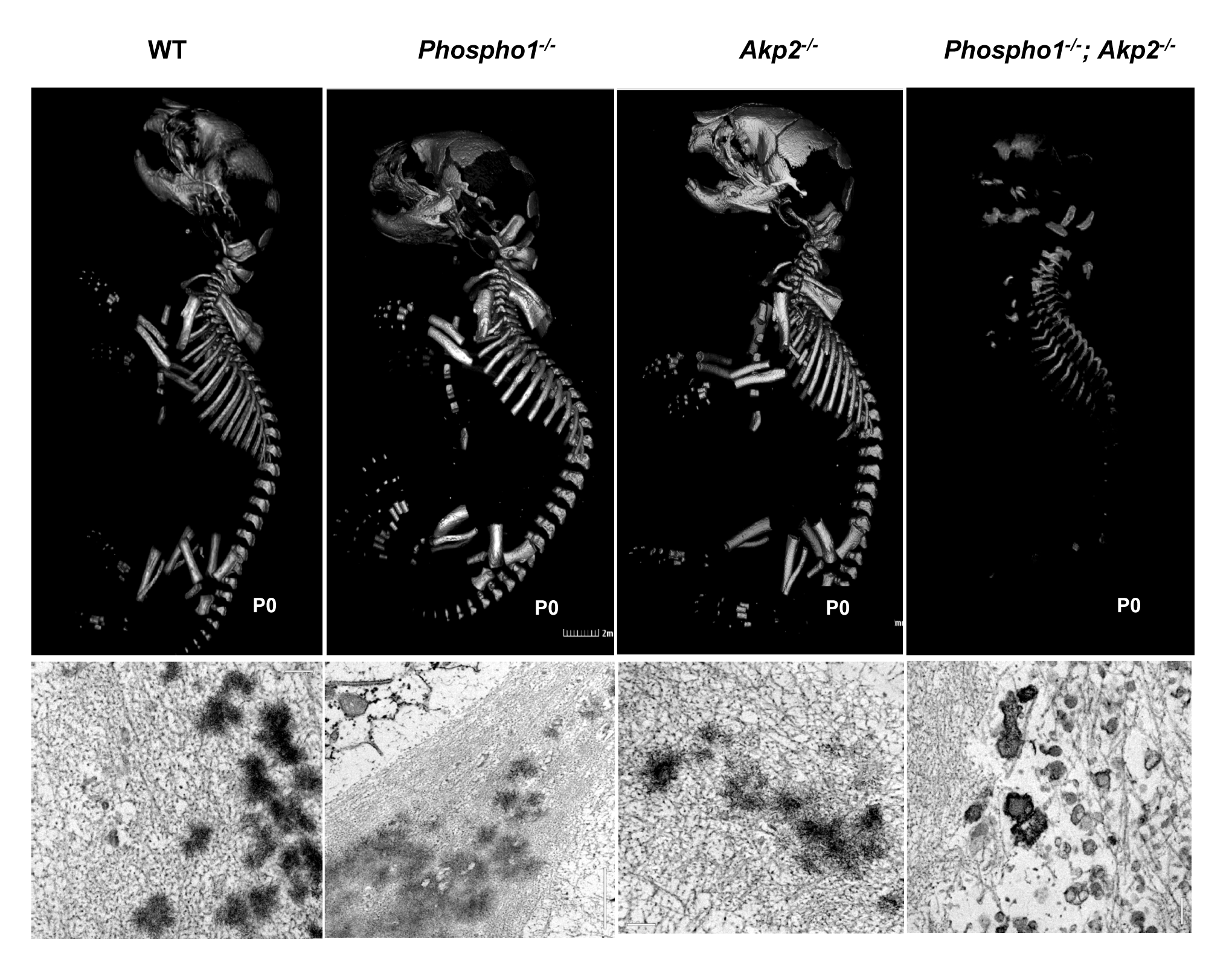

Supplement: Supplementary file 2 [file jbmr0026-0286-SD2.tif]

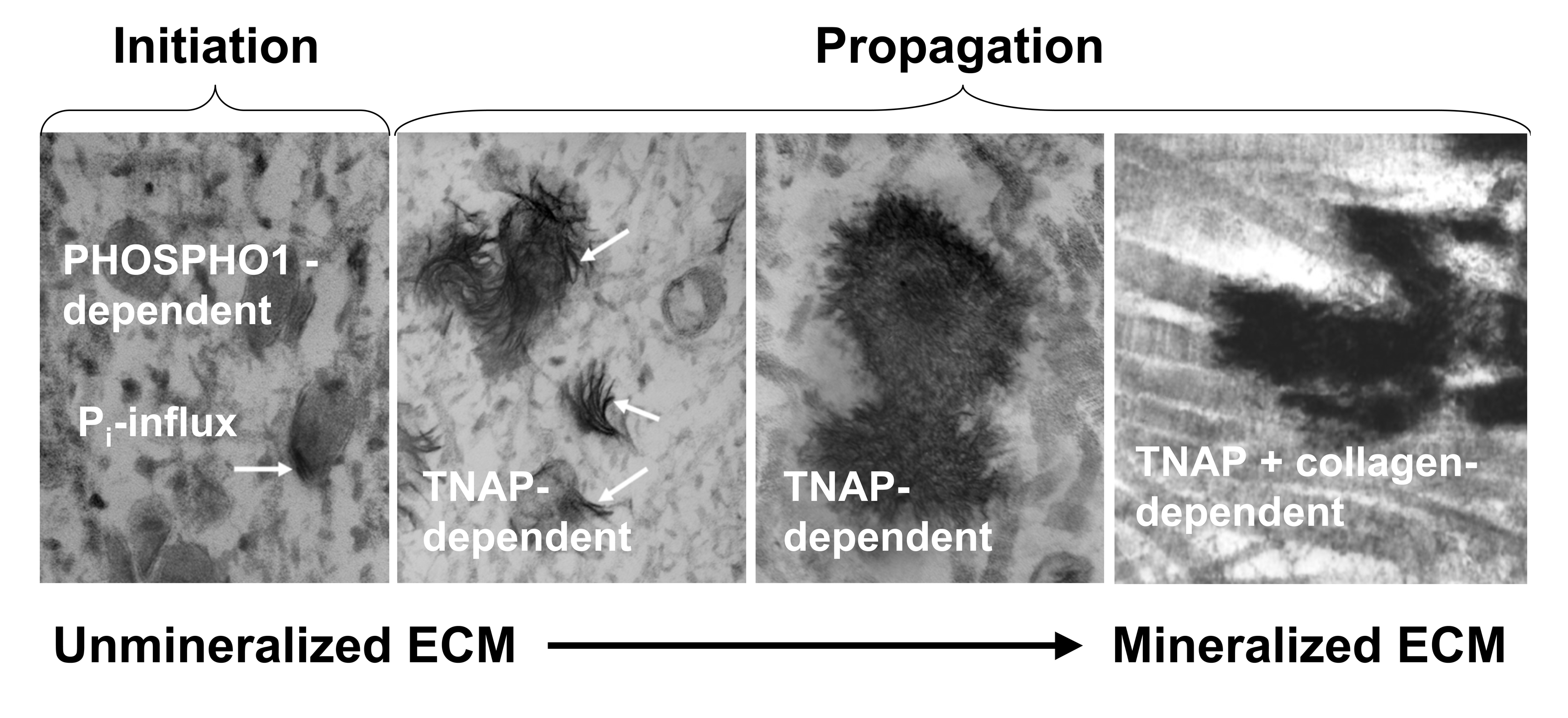

Supplement: Supplementary file 3 [file jbmr0026-0286-SD3.tif]
