# Supplementary material for: Loss of Skeletal Mineralization by the Simultaneous Ablation of PHOSPHO1 and Alkaline Phosphatase Function: A Unified Model of the Mechanisms of Initiation of Skeletal Calcification
Source: J Bone Miner Res. 2010 Aug 3;26(2):286–97. doi: 10.1002/jbmr.195 (PMC3179344; doi:10.1002/jbmr.195)
Supplement: Supplementary file 4 [file jbmr0026-0286-SD4.doc]

**Legends to Supplemental Figures 1 through 3**

**Supplemental Figure 1: *Chondrocyte gene expression changes in Phospho1^-/-^ mice -*** qPCR analysis showed a decrease in *Col2a1* expression in *Phospho1^-/-^* chondrocytes at day 1 (*P* = 0.03), Day 7 (*P* = 0.03) and day 14 (*P* = 0.02) in culture. A similar change was observed in *Aggrecan* expression (day1, *P* = 0.01; day 7, *P* = 0.03; and day 14, *P* = 0.02). *MMP13* expression was not significantly changed on day 1 but decreased on day 7 (*P* = 0.003) and day 14 (*P* = 0.02). *Col10a1* expression was also decreased on day 14 (*P* = 0.008). A slight increase in *Runx2* expression was observed on day 1 cultures (*P* = 0.02) but there was no difference on day 7 and day 14 in *Phospho1^-/-^* chondrocytes as compared to WT cells*.*  Data are represented as mean ± SEM, N = 3, experiments done in triplicates.

**Supplemental Figure 2: *µCT and transmission electron microscopy (TEM) analysis of the calcification in P0 pups.*** *Phospho1^-/-^* P0 pups show deformed long bones and ribs as compared to the WT mice. The only stillborn [*Phospho1^-/-^; Akp2^-/-^*] pup, out of 272 examined, show complete lack of mineralization in the appendicular skeleton, severely reduced mineralization in the axial skeleton including vertebral bodies, the skull and jaw bones. At the TEM level, reduced ECM calcification is apparent in the vertebrae of *Phospho1^-/-^* and *Akp2^-/-^* samples. An increased number of MVs and greatly reduced ECM calcification is observed in the vertebrae of the stillborn [*Phospho1^-/-^; Akp2^-/-^*] pup.

**Supplemental Figure 3: *Unified model of the mechanisms of initiation of skeletal mineralization.*** At the initiation of MV-mediated mineralization (first phase or initiation step), hydroxyapatite (HA) crystals appear inside the MVs favored by P_i_ accumulation resulting from a dual mechanism, i.e. PHOSPHO1-mediated intravesicular production, via degradation of phosphothanolamine and phosphocholine, and transporter-mediated influx of P_i_ produced extravesicularly primarily by TNAP’s ATPase activity or, secondarily in the absence of TNAP, by NPP1’s ATPase activity. Extravesicular mineralization of the extracellular matrix (ECM) (second phase or propagation step) is mainly supported by TNAP’s pyrophosphatase activity, and secondarily by NPP1’s pyrophosphatase activity (in the absence of TNAP) and is driven by the availability of P_i_ and the presence of a collagenous fibrilar scaffold and other non-collagenous ECM proteins. White arrows point to nascent HA crystals inside the MVs or crystals breaking through the MV membranes.
