# Supplementary material for: Loss of Skeletal Mineralization by the Simultaneous Ablation of PHOSPHO1 and Alkaline Phosphatase Function: A Unified Model of the Mechanisms of Initiation of Skeletal Calcification
Source: J Bone Miner Res. 2010 Aug 3;26(2):286–97. doi: 10.1002/jbmr.195 (PMC3179344; doi:10.1002/jbmr.195)
Supplement: Supplementary file 5 [file jbmr0026-0286-SD5.doc]

**Supplemental Table 1:** µCT analysis of trabecular and cortical bones in tibia and femur of WT and *Phospho1^-/-^* mice. The following parameters were measured: bone volume fraction (BV/TV), trabecular thickness (Tr. Th.), trabecular separation (Tr. Sp.), trabecular number (Tr. No.), trabecular pattern formation (Tr. Pf.), structural model index (SMI), degree of anisotropy (DA), cortical porosity (Cor. Por.) and cortical thickness (Cor.Th.).

**Trabecular Bone**

| **Tibia** | **WT** | ***Phospho1^-/-^*** | **P value** |
| --- | --- | --- | --- |
| **BV/TV** | 9.018 ± 1.89 | 11.458 ± 2.54 | 0.052 |
| **Tr. Th.** | 36.113 ± 1.89 | 35.716 ± 2.42 | 0.726 |
| **Tr. Sp.** | 237.761 ± 41.84 | 172.864 ± 17.854 | <0.001 |
| **Tr. No.** | 0.00249 ± 0.0004 | 0.00318 ± 0.0005 | 0.012 |
| **Tr. Pf.** | 0.0306 ± 0.0037 | 0.0305 ± 0.00149 | 0.973 |
| **SMI** | 1.763 ± 0.084 | 1.803 ± 0.011 | 0.461 |
| **DA*** | 2.007 | 1.753 | 0.259 |

| **Femur** | **WT** | ***Phospho1^-/-^*** | **P value** |
| --- | --- | --- | --- |
| **BV/TV** | 14.921 ± 2.541 | 15.847 ± 3.142 | 0.556 |
| **Tr. Th.** | 41.335 ± 2.16 | 39.108 ± 4.038 | 0.223 |
| **Tr. Sp.** | 179.413 ± 23.154 | 157.149 ± 17.258 | 0.064 |
| **Tr. No.** | 0.00361 ± 0.0006 | 0.00404 ± 0.00062 | 0.199 |
| **Tr. Pf.** | 0.0207 ± 0.00261 | 0.0215 ± 0.00455 | 0.700 |
| **SMI** | 1.994 ± 0.0829 | 2.007 ± 0.102 | 0.791 |
| **DA*** | 2.252 | 1.928 | 0.044 |

**Cortical Bone**

| **Tibia** | **WT** | ***Phospho1^-/-^*** | **P value** |
| --- | --- | --- | --- |
| **Cor. Por.** | 62.12 ± 4.4 | 64.64 ± 5.35 | 0.313 |
| **Cor. Th.** | 172.77 ± 8.62 | 154.06 ± 14 | 0.009 |

| **Femur** | **WT** | ***Phospho1^-/-^*** | **P value** |
| --- | --- | --- | --- |
| **Cor. Por.** | 71.54 ± 5.8 | 74.05 ± 5.34 | 0.04 |
| **Cor. Th.** | 158.88 ± 8.11 | 165.53 ± 17.68 | 0.31 |
