# Supplementary material for: Loss of Skeletal Mineralization by the Simultaneous Ablation of PHOSPHO1 and Alkaline Phosphatase Function: A Unified Model of the Mechanisms of Initiation of Skeletal Calcification
Source: J Bone Miner Res. 2010 Aug 3;26(2):286–97. doi: 10.1002/jbmr.195 (PMC3179344; doi:10.1002/jbmr.195)
Supplement: Supplementary file 6 [file jbmr0026-0286-SD6.doc]

**Supplemental Table 2:** Serum biochemistry of 10-day-old and 1-year-old WT and *Phospho1^-/-^* mice (N = 4 per group).

| **Serum components** | **10- day- old** | | **1- year- old** | |
| --- | --- | --- | --- | --- |
|  | **WT** | ***Phospho1^-/-^*** | **WT** | ***Phospho1^-/-^*** |
| **Glucose (mG/dL)**  **BUN (mG/dL)**  **Creatinine (mG/dL)**  **Albumin (G/dL)**  **Globulin (G/dL)**  **Total Protein (G/dL)**  **Total Bilirubin (mG/dL)**  **Sodium (mmol/L)**  **Potassium (mmol/L)**  **Calcium (mG/dL)**  **Phosphorus (mG/dL)** | 149.8 ± 6.16  21.75 ± 3.12  0.2 ± 0  2.28 ± 0.1  0.98± 0.5  3.23 ± 0.06  0.4 ± 0.04  133.8 ± 0.48  5.9 ± 0.5  11.5 ± 0.19  10.43 ± 0.64 | 153.3 ± 6.44  37.5 ± 3.23  0.23 ± 0.03  1.95 ± 0.9  1.4 ± 0.06  3.33 ± 0.09  0.38 ± 0.03  136.8 ± 0.66  6.0 ± 0.6  11.4 ± 0.6  13.55 ± 0.75 | 95.2 ± 9.3  20 ± 1.8  0.2 ± 0  3.7 ± 0.03  2.4 ± 0.15  6.2 ± 0.014  0.3 ± 0.03  162 ± 1.15  6.9 ± 0.48  10.5 ± 0.15  6.0 ± 0.84 | 95 ± 23.7  15.6 ± 1.9  0.2 ± 0  3.8 ± 0.13  2.5 ± 0.17  6.3 ± 0.09  0.3 ± 0.07  161.3 ± 1.3  6.5 ± 0.5  10.3 ± 0.27  5.3 ± 0.62 |
