# Supplementary material for: Loss of Skeletal Mineralization by the Simultaneous Ablation of PHOSPHO1 and Alkaline Phosphatase Function: A Unified Model of the Mechanisms of Initiation of Skeletal Calcification
Source: J Bone Miner Res. 2010 Aug 3;26(2):286–97. doi: 10.1002/jbmr.195 (PMC3179344; doi:10.1002/jbmr.195)
Supplement: Supplementary file 7 [file jbmr0026-0286-SD7.doc]

**Supplemental Table 3:** Numbers and genotypes of pups born from (A) [*Phospho1^+/-^*; *Akp2^+/-^*] x [*Phospho1^+/-^*; *Akp2^+/-^*] and (B) [*Phospho1^-/-^*; *Akp2^+/-^*] x [*Phospho1^-/-^*; *Akp2^+/-^*] matings.

A

|  | **Expected %** | **# of mice born** | **Actual %** |
| --- | --- | --- | --- |
| ***Phospho1^+/-^*; *Akp2^+/-^*** | 25 | 64 | 28.4 |
| ***Phospho1^+/+^*; *Akp2^+/-^*** | 12.5 | 20 | 8.9 |
| ***Phospho1^-/-^*; *Akp2^+/-^*** | 12.5 | 13 | 5.8 |
| ***Phospho1^+/-^*; *Akp2^+/+^*** | 12.5 | 49 | 21.8 |
| ***Phospho1^+/-^*; *Akp2^-/-^*** | 12.5 | 19 | 8.4 |
| ***Phospho1^+/+^*; *Akp2^+/+^*** | 6.25 | 17 | 7.6 |
| ***Phospho1^-/-^*; *Akp2^+/+^*** | 6.25 | 19 | 8.4 |
| ***Phospho1^+/+^*; *Akp2^-/-^*** | 6.25 | 5 | 2.2 |
| ***Phospho1^-/-^*; *Akp2^-/-^*** | 6.25 | 1 (stillborn) | 0.4 |
|  |  | Total = 225 |  |

B

|  | **Expected %** | **# of mice born** | **Actual %** |
| --- | --- | --- | --- |
| ***Phospho1^-/-^*; *Akp2^+/+^*** | 25 | 31 | 66.0 |
| ***Phospho1^-/-^*; *Akp2^+/-^*** | 50 | 16 | 34.0 |
| ***Phospho1^-/-^*; *Akp2^-/-^*** | 25 | 0 | 0 |
|  |  | Total = 47 |  |
