# Supplementary material for: Loss of Skeletal Mineralization by the Simultaneous Ablation of PHOSPHO1 and Alkaline Phosphatase Function: A Unified Model of the Mechanisms of Initiation of Skeletal Calcification
Source: J Bone Miner Res. 2010 Aug 3;26(2):286–97. doi: 10.1002/jbmr.195 (PMC3179344; doi:10.1002/jbmr.195)
Supplement: Supplementary file 8 [file jbmr0026-0286-SD8.doc]

**Supplemental Text:**

RNA was extracted using RNAeasy Pus Kit (Qiagen, Valencia, CA, USA). Specific RNA transcripts (mRNA) were quantified by real-time PCR using dual-labeled hydrolysis probes (FAM-TAMRA). The primers and probe sequences were obtained from **Eurogentec North America, Inc.** (San Diego, CA) and are as follows:

*18S*

F: 5’-CGGCTACCACATCCAAGGAA-3’

R: 5’-GCTGGAATTACCGCGGCT-3’

Probe: 5’-TGCTGGCACCAGACTTGCCCTC-3’

*Phospho1*

F: 5’-CTATCTAGGGACGGCAGGAT-3’

R: 5’-TCTCATCGAAGTCGAAGGTG-3’

Probe: 5’-CGCGCCTCGCTTCCTCCT-3’

*Akp2*

F: 5’-CTGCCACTGCCTACTTGTGT-3’

R: 5’-GATGGATGTGACCTCATTGC-3’

Probe: 5’-CTGTGGCTGCGCTCACTCCC-3’

*Enpp1*

F: 5’-CTTCCTGTATCTCGCTGCCT-3’

R: 5’-TCTGAGATTGAGGCACATCC-3’

Probe: 5’-CATGAGTCCACGCCATGGCA-3’

*Ank*

F: 5’-GTTCACCTTTGTCTGCATGG-3’

R: 5’-CAGAGTTCTGCAAAGGCAAA-3’

Probe: 5’-CGCTGTCGCTGACGCTCTGTT-3’

*MMP13*

F: 5’-AAGATGTGGAGTGCCTGATG-3’

R: 5’-CAGGAGTATAGTTCACAATCCTGTAAG-3’

Probe: 5’-TCCCTAGAACACTCAAATGGTCCCAAA-3’

*Col10a1*

F: 5’-CCCATACGCCATAAAGGATT-3’

R: 5’-GTCCAGGACTTCCATAGCCT-3’

Probe: 5’-CTGGTCCACCAGGCCCAACC-3’

*Aggrecan*

F: 5’-TGCCTTGGACACTTTCACAT-3’

R: 5’-TGGTCGATCTCACACAGGTT-3’

Probe: 5’-CCCTCTGTAGCTCGGAAGGCATAAGC-3’

*Col2a1*

F: 5’-AGGTTCACATACACTGCCCTG-3’

R: 5’-CGATGACGGTCTTGCCCCACTT-3’

Probe: 5’-AGGATGGCTGCACGAAACACACTGG-3’

*Runx2*

F: 5’-AGTAGCCAGGTTCAACGATCTGA-3’

R: 5’-GGACCGTCCACTGTCACTTTAATA-3’

Probe: 5’-CCGGAGCGGACGAGGCAAGAGTT-3’
